# Early Discharge in Low-Risk Patients Hospitalized for Acute Coronary Syndromes: Feasibility, Safety and Reasons for Prolonged Length of Stay

**DOI:** 10.1371/journal.pone.0161493

**Published:** 2016-08-23

**Authors:** Marie-Eva Laurencet, François Girardin, Fabio Rigamonti, Anne Bevand, Philippe Meyer, David Carballo, Marco Roffi, Stéphane Noble, François Mach, Baris Gencer

**Affiliations:** 1 Internal Medicine Division, Department of Medicine, Geneva University Hospital, Geneva, Switzerland; 2 Division of Clinical Pharmacology and Toxicology, Department of Anesthesiology, Clinical Pharmacology and Toxicology Intensive Care, Geneva University Hospitals and University of Geneva, Geneva, Switzerland; 3 Medical Direction, Geneva University Hospitals and University of Geneva, Geneva, Switzerland; 4 Cardiology Division, Department of Medicine, Geneva University Hospital, Geneva, Switzerland; GERMANY

## Abstract

**Introduction:**

Length of hospital stay (LHS) is an indicator of clinical effectiveness. Early hospital discharge (≤72 hours) is recommended in patients with acute coronary syndromes (ACS) at low risk of complications, but reasons for prolonged LHS poorly reported.

**Methods:**

We collected data of ACS patients hospitalized at the Geneva University Hospitals from 1st July 2013 to 30th June 2015 and used the Zwolle index score to identify patients at low risk (≤ 3 points). We assessed the proportion of eligible patients who were successfully discharged within 72 hours and the reasons for prolonged LHS. Outcomes were defined as adherence to recommended therapies, major adverse events at 30 days and patients' satisfaction using a Likert-scale patient-reported questionnaire.

**Results:**

Among 370 patients with ACS, 255 (68.9%) were at low-risk of complications but only 128 (50.2%)were eligible for early discharge, because of other clinical reasons for prolonged LHS (e.g. staged coronary revascularization, cardiac monitoring) in 127 patients (49.8%). Of the latter, only 45 (35.2%) benefitted from an early discharge. Reasons for delay in discharge in the remaining 83 patients (51.2%) were mainly due to delays in additional investigations, titration of medical therapy, admission or discharge during weekends. In the early discharge group, at 30 days, only one patient (2.2%) had an adverse event (minor bleeding), 97% of patients were satisfied by the medical care.

**Conclusion:**

Early discharge was successfully achieved in one third of eligible ACS patients at low risk of complications and appeared sufficiently safe while being overall appreciated by the patients.

## Introduction

The length of hospital stay (LHS) has considerably decreased with the improvement of therapies and implementation of evidence-based therapies in patients with acute coronary syndromes (ACS).[1] According to European Society of Cardiology (ESC) guidelines for the management of ST-segment elevation myocardial infarction (STEMI), early discharge (within approximately 72 hours) may be considered in selected patients at low risk of complications, if early rehabilitation and adequate follow-up have been planned (class IIb, level of evidence B).[2] In this regard, the Zwolle index score has been recommended as a clinical tool to detect patients at low risk of complications who can safely benefit from an early hospital discharge.[3] The Zwolle index score include: Killip class (1–4), TIMI flow grade following angioplasty (1–4), age (60 years), three-vessels diseases, anterior infarction and ischemic time (</> 4 hours). A pilot study with 54 patients, reported that early discharge was potentially feasible and safe in ACS patients, although the sample size was limited (54 patients).[4] Observational data suggest that early discharge among ACS patients is poorly implemented in clinical practice, with less than 26% of eligible patients discharged within 4 days. [5]

Furthermore, the reasons of prolonged LHS in low-risk ACS patients are poorly documented. Large administrative data suggest important variations in LHS for ACS across countries and health care systems. For example, financial incentives in the United States have been associated with a shortening of LHS. In an era of growing economic pressures and limited health care resources, the Swiss health care deciders introduced the DRG (Diagnosis Related Groups) framework in January 2012, based on a flat flees financing system, aiming at improving efficiency, transparency, benchmarking, quality, responsibility and comparability between hospitals whilst containing global health care costs. Of note, DRG is a financial incentive to improve clinical effectiveness.[6] The aim of this study was to assess the feasibility and safety of an early discharge (≤ 72 hours) in low-risk ACS patients hospitalized at the Geneva University Hospitals and to document the reasons for prolonged LHS in low-risk patients.[7]

## Methods

### Study population

We analyzed prospectively collected data of patients included in the SPUM-ACS (Special Program University Medicine—Acute Coronary Syndrome, NCT01075867) cohort at the Geneva University Hospitals from 1^st^ July 2013 to 30^th^ June 2015.[8–10] Inclusion criteria were patients older than 18 years old, and diagnosis of ACS complemented with an angiography. ACS was defined as symptoms compatible with angina (chest pain, dyspnea) and one of the following criteria: positive troponin value, ST-segment elevation or depression, T-wave inversion, changes in ECG readings.

### Role of the Ethics Committee and Source of Funding

The study protocol was approved by the institutional review board of all participating centers; namely, the Ethics Committee on Clinical Research of the University of Lausanne, the Ethics Committee of the Department for Internal Medicine and Community Medicine of the University Hospital of Geneva, the Cantonal Ethics Committee (KEK) of the Canton of Bern, and the Cantonal Ethics Committee (KEK) of the Canton of Zurich. All patients provided written, informed consent.

### Zwolle index score and reasons for prolonged hospital stay

We classified eligibility for early discharge using the Zwolle index score. The Zwolle index score is a validated and recommended score to identify patients at low risk of complications after ACS, stratifying patients into 2 groups: low risk (≤ 3 points) and high risk (> 3 points).[2,3] If a patient was eligible for early discharge, we collected data of pre-specified conditions (medical and non-medical) requiring prolonged LHS, additional coronary lesion requiring staged revascularization during the same hospitalization, delayed transfer to another hospital (because of place problem in that hospital), cardiac monitoring, post- percutaneous coronary intervention (PCI) myocardial infarction, pericardial effusion, major bleeding events (defined by BARC 3 or 5), left ventricular ejection fraction (LVEF) ≤40%, cardiac thrombus, acute renal failure, incapacities in physical mobility and refusal to attend cardiac rehabilitation after hospital discharge. During the hospital stay, the cardiology division of the Geneva University Hospitals in case of eligibility for an early discharge (Zwolle index score ≤ 3 points without exclusion criteria), collected reasons for prolonged hospital stay based on previously published criteria: (1) patient criteria (e.g. anxiety, social), (2) organizational related criteria (delay in complementary exams, hospital admission during week-end), (3) medical related criteria (additional time for titration of medication).

### Length of hospital stay

We assessed LHS based on hospital administrative data measuring the data and time of hospital admission (defined as the entry to the emergency department or directly to the cardiac catherization laboratory) and the date and time of hospital discharge (defined as discharge from the hospital ward). Administrative data are used as a quality indicator for DRG-based reimbursement, as well as in registry-based observational studies. Successful early discharge was based on the Zwolle index score and ESC guidelines for ACS, and was defined as discharge occurring within 72 hours following hospitalization. Discharge beyond 72 hours was qualified as standard discharge.

### Safety evaluation

For early discharge patients, major adverse cardiovascular events (MACE) were systematically collected at 30 days, either by telephone from the patient or on the basis of medical records from the hospital or treating physician. MACE were defined as all-cause death, cardiovascular death, myocardial infarction, cerebrovascular events, unplanned coronary revascularization, bleeding events and hospitalization for unstable angina. All MACE were adjudicated by 2 certified cardiologists. We also collected data on the recommended discharge treatments: aspirin, P2Y_12_ inhibitors (clopidogrel, prasugrel, ticagrelor), statins, beta-blockers and angiotensin-converting enzyme (ACE) inhibitors or angiotensin II receptor blockers (ARB).

### Patient satisfaction

Using a *Lickert*-scale, we measured patient satisfaction for LHS. As done in a previous study assessing patient satisfaction for outpatient treatment of pulmonary embolism patients weasked the following questions at 30 days following hospital discharge: «*What is your global appreciation of the medical care you received during your hospital stay*? », «*Were you satisfied by the medical instruction provided at the hospital*? ». [11] In addition, the following questions were asked, depending on whether patients had benefitted from early discharge «*Would you have preferred a longer hospital stay*? »; or had stayed for longer than 72 hours: «*Would you have preferred to be discharged earlier*? »

### Statistical analysis

**Fig 1:**
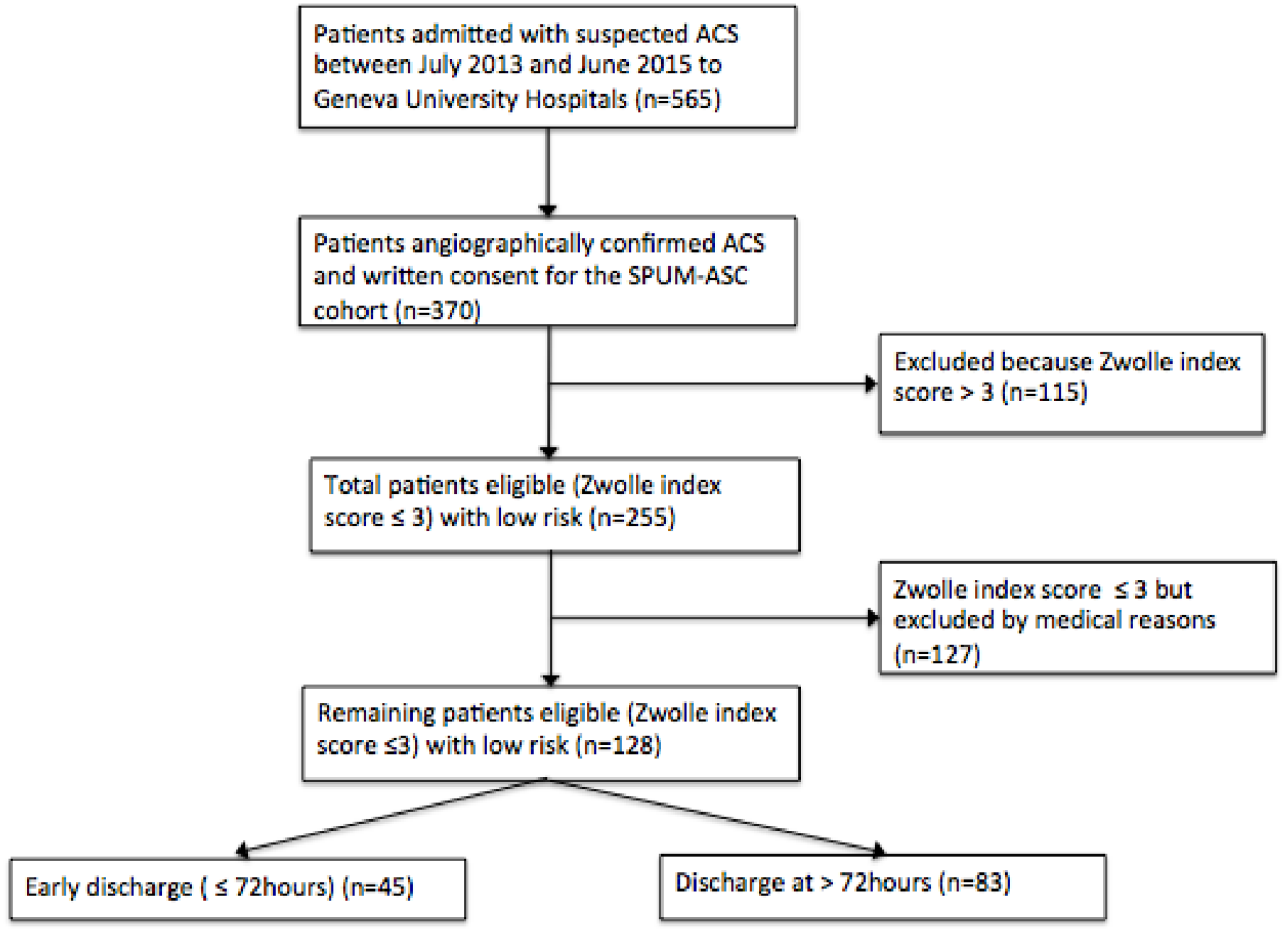
Flowchart of the inclusion process of ACS patients admitted in Geneva University Hospitals between July 2013 and June 2015.

**Fig 2:**
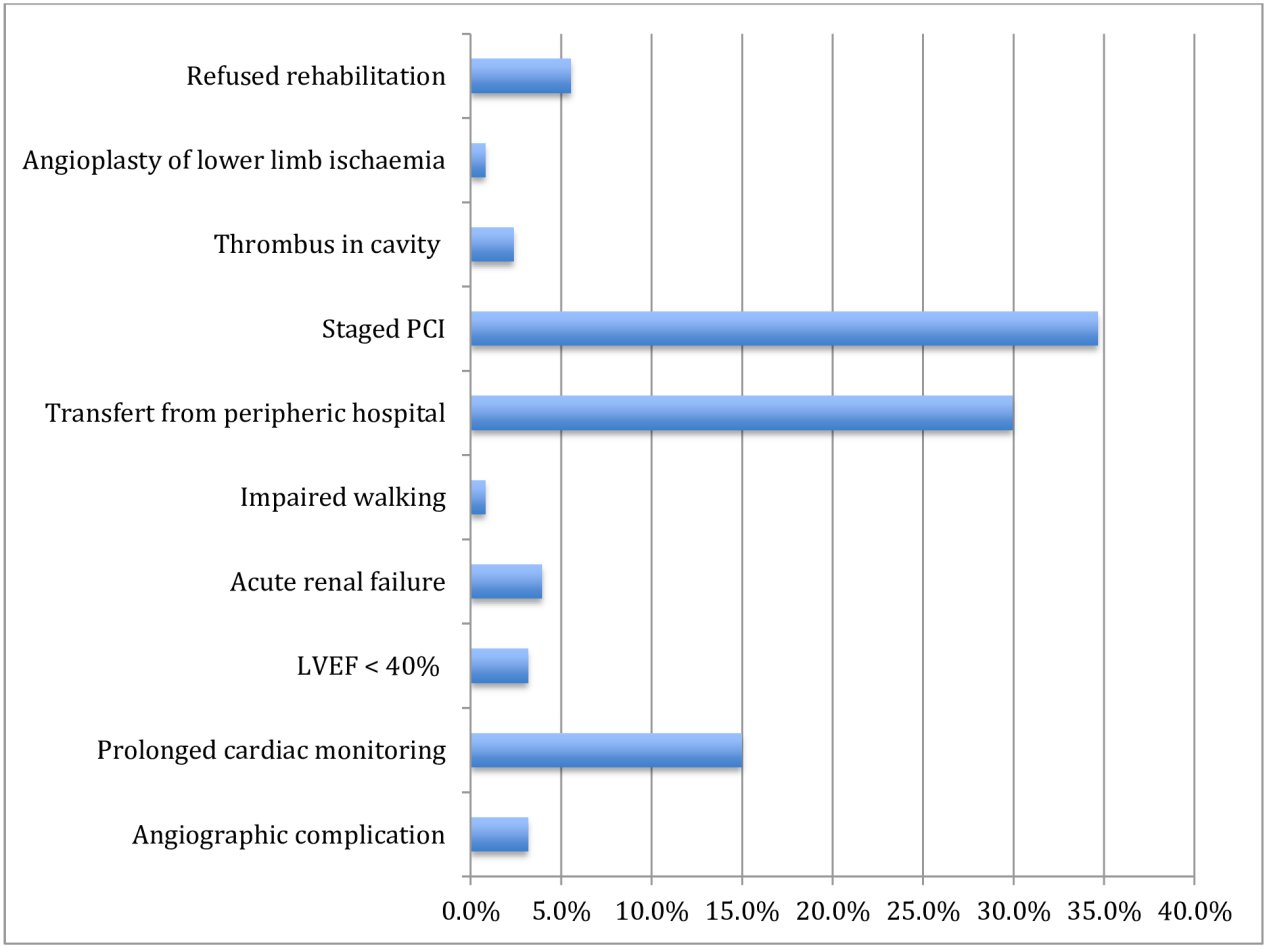
Documented clinical reasons for not applying early discharge in 127 ACS patients with a Zwolle index score ≤ 3 points (at low risk of complications). Abbreviations: LVEF: Left ventricular ejection fraction, PCI: percutaneous coronary intervention.

**Fig 3:**
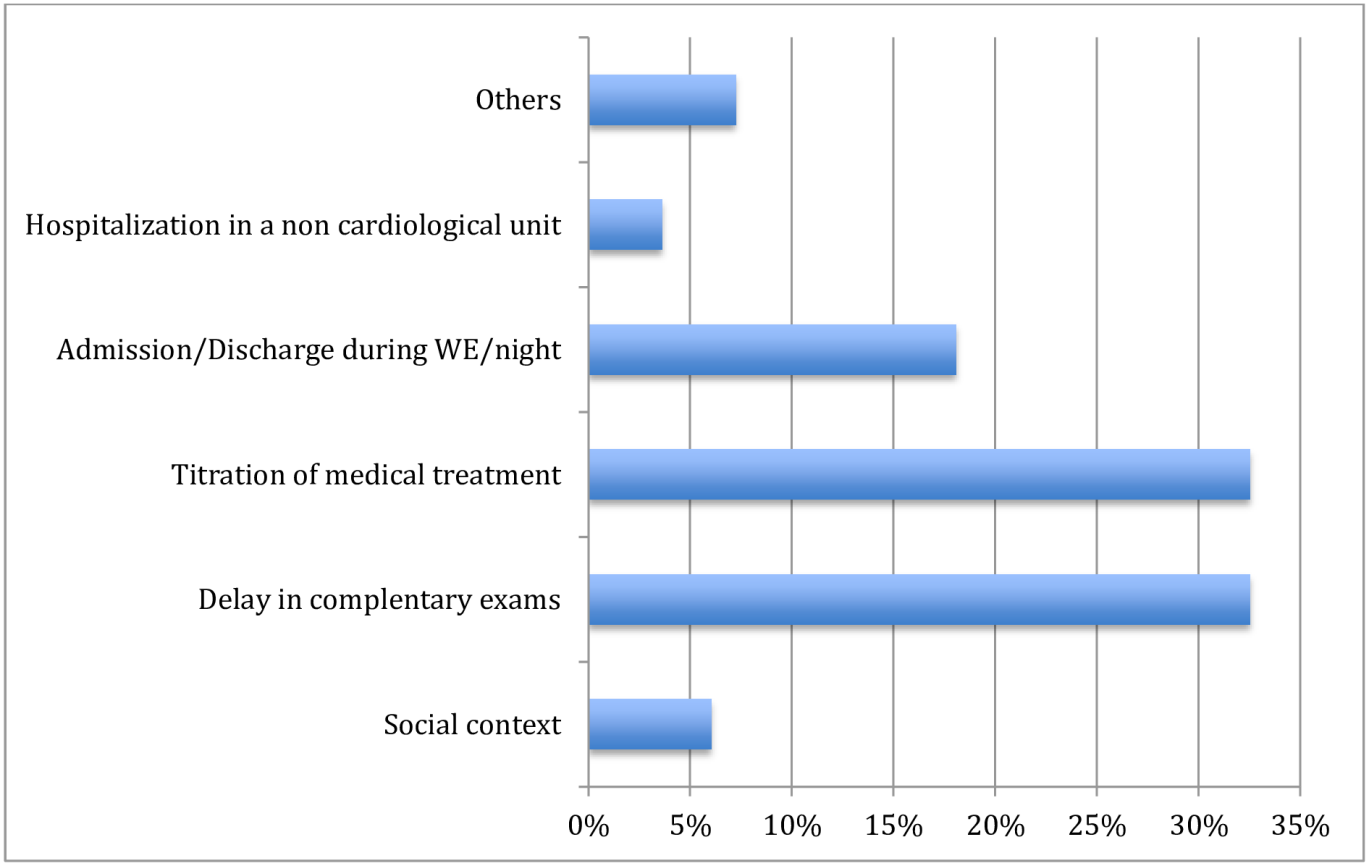
Documented reasons for prolongation of hospitalization in 83 patients with a Zwolle index score ≤ 3 points (at low risk of complications) and without clinical reasons. Abbreviatsions: WE, weekend.

The flowchart of the inclusion process using the STROBE criteria is detailed in Fig 1. Patient characteristics are described in Fig 2, according to their eligibility for early discharge, using mean and standard deviations for continuous variables and frequencies for categorical variables. Reasons for prolonged hospital stay, discharge treatment, MACE and patient satisfaction are presented in Fig 3, using frequencies in both groups (early vs. standard discharge). The primary outcome was to evaluate the feasibility and the safety of early discharge in low-risk (Zwolle score index ≤ 3) patients after ACS and successful PCI. The secondary outcome was to determine the reasons of prolonged hospitalization, the rate of complications and patient satisfaction at 30 days. We also compared treatments in the two groups, at discharge and at 1 year. Data were entered into a FileMaker Pro dataset and the level of significance was set at a p value < 0.05.

## Results

### Baseline characteristics

Among 370 patients included in the analysis, 255 (68.9%) were eligible for early discharge (Zwolle index score ≤ 3 points) compared to 115 patients who had a Zwolle index score > 3 points (Fig 1 and S1 Fig). The mean age of patients eligible for early discharge was younger (57.4 ± 26.7 vs 68.9 ±9.8, P < 0.001) and with more comorbidities, such as preexisting hypertension than patients not eligible for early discharge (Table 1). ACS subtypes were similar in both groups, but patients not eligible for early discharge underwent less PCI revascularization (88.7% vs 95.7% P<0.012). The peak troponin value (ng/l) was also lower in early discharge patients (0.38+/-3.57 vs 0.67 +/-4.0, p = 0.487), whereas LVEF (%) was similar across groups (55 1/-15 vs 55 +/-13.8 p = 1.000).

**Table 1:** Baseline characteristics of 255 ACS patients eligible for early discharge (Zwolle index score < 3 points) and 115 ACS patients not eligible for early discharge (Zwolle index score ≥3 points).

CHARACTERISTICS	Eligible	Not eligible	p value
	n = 255	n = 115	
**Age (year)**	57.36+/-11.8	68.6+/-9.8	*p = 0*.*001*
**Sex (male)**	82.0% (n = 209)	81.7% (n = 94)	p = 0.96
**BMI (kg/m2)**	26.7 +/- 4.0	27.4 +/-4.5	p = 0.136
**Working status**			
* Full/part time*	58.8%(n = 150)	33.0% (n = 38)	*p = 0*.*000003*
* No employement/retired*	39.6% (n = 101)	66.1% (n = 76)	
**Educational status**			
* University/High Scool*	45.9% (n = 117)	36.5% (n = 42)	p = 0.100
* Vocational school*	50.6% (n = 129)	59.1% (n = 68)	
**Cariovascular risk factors**			
* Diabetes*	11.4%% (n = 29)	22.6% (n = 26)	*p = 0*.*005*
* Hyperlipidemia*	59.2% (n = 151)	66.1% (n = 76)	p = 0.209
* Hypertension*	44.7% (n = 114)	62.6% (n = 72)	*p = 0*.*001*
* Current smoker*	47.5% (n = 121)	30.4% (n = 35)	p = 0.685
**Previous MI**	6.3% (n = 16)	8.7% (n = 10)	p = 0.399
**Previous PCI**	8.6% (n = 22)	13.0% (n = 15)	p = 0.190
**Previous CABG**	161% (n = 4)	7.8% (n = 9)	*p = 0*.*002*
**Index STEMI**	49.4% (n = 126)	46.1% (n = 53)	p = 0.678
**Index NSTE-ACS**	47.5% (n = 121)	48.7% (n = 56)	
**Door-to-ballon time (min)**	193.5 +/- 644.75		
**Index revascularisation**			
* PCI with stent*	95.7% (n = 244)	88.7% (n = 102)	*p = 0*.*012*
* Conservative*	4.3% (n = 11)	10.4% (n = 12)	
* CABG*	0.0% (n = 0)	0.9% (n = 1)	
**Peak troponine value (ng/l)**	0.38+/- 3.57	0.67+/-4.0	p = 0.487
**LVEF (%)**	55+/-15	55+/-13.8	p = 1.000

LVEF: left ventricular ejection fraction, PCI,: percutaneous coronary intervention, CVRF: cardiovascular risk factors, BMI: body mass index, STE-ACS: ST elevation-Acute Coronary Syndrome, NSTE-ACS: Non ST elevation- Acute Coronray Syndrome, CABG: coronary artery bypass grafting. Missing values for patients eligible for early discharge: 4 for workings status, 2 for martial status, 9 for educational status, 9 for LVEF, 2 for BMI, 8 for NSTE-ACS index, 123 for door-to-balloon. Missing values for patients not eligible for early discharge: 1 for workings status, 5 for educational status, 13 for LVEF, 6 for NSTE-ACS index

Among 255 patients who were eligible for early discharge, 127 had additional criteria for prolonged hospital stay (F 1); the leading causes for prolonged hospitalization were a staged procedure (34.6%) with a second coronary lesion requiring PCI during the same hospitalization, and cardiac monitoring (15.0%; Fig 2). A further 29.9% of patients were transferred back to the peripheral hospital from which they had been referred. Among the remaining 128 patients, only 45 (35.1%) benefitted from an early discharge while 83 were discharged beyond 72 hours. In both groups, no significant differences were found for age, sex, working status, marital status, cardiovascular risk factors, revascularization strategies and LVEF. However, patients with early discharge group were more likely to have a non-ST elevation ACS compared to standard discharge group (P = 0.068) (Table 2). The most common reported reasons for prolonged hospitalization were additional time for titration of medications (33%), delay in complementary exams such as echocardiography (33%), or admission/discharge during the weekends/nights (18%). There were no differences in discharge medications between the early and standard discharge groups: aspirin 97.5% vs 97.6%, statins 95.6% vs 95.2%, beta-blockers 86.7% vs 85.5% and ACEI/ARB 93.3% vs 86.7%. Attendance to cardiac rehabilitation was 87.5% vs. 83.3% (Table 3).

**Table 2:** Characteristics of 45 early discharge (≤ 72 hours) ACS patients and 83 standard discharge ACS patients at low risk of complications (Zwolle index score < 3 points).

CHARACTERISTICS	Early discharge n = 45	Standard discharge n = 83	p value
**Age (year)**	54.7+/-8.6	57.8 +/-11.7	p = 0.121
**Sex (male)**	88.9% (n = 40)	81.9% (n = 68)	p = 0.300
**BMI (kg/m2)**	27 +/-4.2	26.5 +/-3.6	p = 0.481
**Working status**			
*Full/part time*	66.7% (n = 30)	57.8% (n = 48)	p = 0.328
*No employement/retired*	33.3% (n = 15)	42.2% (n = 35)	
**Educational status**			
*University/High Scool*	48.9% (n = 22)	41.0% (n = 34)	p = 0.491
*Vocational school*	51.1% (n = 23)	55.4% (n = 46)	
**Cardiovascular risk factor**			
*Diabetes*	8.9% (n = 4)	10.8% (n = 9)	p = 0.727
*Hyperlipidemia*	53.3% (n = 24)	60.2% (n = 50)	p = 0.450
*Hypertension*	46.7% (n = 21)	45.8% (n = 38)	p = 0.923
*Current smoker*	51.1% (n = 23	51.8% (n = 43)	p = 0.940
**Previous MI**	6.7% (n = 3)	8.4% (n = 7)	p = 0.722
**Previous PCI**	6.7% (n = 3)	9.6% (n = 8)	p = 0.567
**Previous CABG**	0.0% (n = 0)	1.2% (n = 1)	p = 0.460
**Index STEMI**	35.6% (n = 16)	51.8% (n = 43)	p = 0.068
**Index NSTE-ACS**	64.4% (n = 29)	47.0% (n = 39)	
**Door-to-balloon time (min)**	209+/-711	159.5 +/- 571	p = 0.670
**Index revascularisation**			
*PCI*	100.0% (n = 45)	98.8% (n = 82)	p = 0.546
*Conservative*	0.0% (n = 0)	1.2% (n = 1)	
*CABG*	0.0% (n = 0)	0.0% (n = 0)	
**Peak troponine value (ng/l)**	0.39+/-1.8	0.68+/-4.0	p = 0.646
**LVEF (%)**	55+/-15	55 +/-15	p = 1.000

LVEF: left ventricular ejection fraction, PCI: percutaneous coronary intervention, CVRF: cardiovascular risk factors, BMI: body mass index, STE-ACS: ST elevation-Acute Coronary Syndrome, NSTE-ACS: Non ST elevation- Acute Coronary Syndrome, CABG: coronary artery bypass grafting.Missing values for standard care: 3 for educational status, 2 for LVEF, 1 for NSTE-ACS index, 1 for door-to-balloon time.

**Table 3:** Adherence to recommended therapies in 45 early discharge (≤ 72 hours) ACS patients and 83 standard discharge ACS patients at low risk of complications (Zwolle index score < 3 points) at time of hospital discharge.

MEDICATIONS	Early discharge n = 45	Standard discharge n = 83	p value
**Aspirin**	97.5% (n = 44)	97.6% (n = 81)	p = 0.947
**P2Y12 inhibitors**			
*Clopidogrel*	24.4% (n = 11)	21.7% (n = 18)	
*Prasugrel*	35.6% (n = 16)	43.4% (n = 36)	p = 0.658
*Ticagrelor*	35.6% (n = 16)	30.1% (n = 25)	
**Beta-blocker**	86.7% (n = 39)	85.5% (n = 71)	p = 0.989
**ACE/ARBs**	93.3% (n = 42)	86.7% (n = 72)	*p = 0*.*027*
**Statins**	95.6% (n = 43)	95.2% (n = 79)	p = 0.828
**Cardiac rehabilitation**	87.5% (n = 35)	83.3% (n = 65)	p = 0.551

ACE: angiotensin converting enzyme, ARBs: angiotensin II receptor blockers

Missing values for early discharge: 5 for rehabilitation at discharge

Missing values for standard discharge: 1 for standard care at discharge in all groups, 5 for rehabilitation at discharge

### Safety at 30 days

The rate of MACE at 30 days was 2.2% (1/45) in the early discharge group and 3.6% in the standard discharge group (3/83). In the early discharge group, we observed one minor bleeding event and one case of an emergency visit for non-cardiac chest pain. In patients who were discharged after 72 hours, we observed one recurrent myocardial infarction, one unplanned revascularization and one bleeding event (Table 4).

**Table 4:** Clinical events at 30 days in 45 ACS patients with early discharge and in 83 ACS patients with standard discharge at low risk of complications (Zwolle index score < 3 points).

Clinical events at 30 days	Early discharge N = 45	Standard discharge N = 83	p value
Total	2.2% (n = 1)	3.6% (n = 3)	p = 0.67
Death	0	0	
Myocardial infarction	0	1	
Unplanned revascularization	0	1	
Major bleeding (BARC 3 or 5)	0	0	
Minor bleeding (BARC 2)	1	1	

All clinical events were adjudicated by a panel of three certified cardiologists blinded to the discharge strategy. 8 missing values for clinical events

### Patient satisfaction

In the early discharge group, 73% (33/45) of patients were fully satisfied with the LHS, 18% (8/45) were almost satisfied, and 2% (2/45) patients would have wanted to stay longer than 72 hours (Table 5). In the standard discharge group, 30% (25/83) of patients would have wanted a shorter hospital stay while 22% (18/83) were not really satisfied by the LHS. In the early discharge group, 84% (38/45) of patients were very satisfied with the medical care provided and 91% (41/45) were very satisfied or satisfied by the medical instructions that were delivered.

**Table 5:** Evaluation of patient satisfaction among 45 early discharge (≤ 72 hours) ACS patients and 83 standard discharge ACS patients at low risk of complications (Zwolle index score < 3 points).

**Evaluation of care at 30 days**	**Early discharge**	**Standard discharge**	**p value**
**Very satisfied**	84% (N = 38)	77% (n = 64)	
**Satisfied**	13% (N = 6)	17% (n = 14)	
**Undecided**	2% (N = 1)	6% (n = 5)	p = 0.331
**Unsatisfied**	0% (n = 0)	0% (n = 0)	
**Very unsatisfied**	0% (n = 0)	0% (n = 0)	
**If discharged** ≤ **72 hours, preference for a later discharge?**		**Patients, % (n = 45)**	
**Yes**		2% (n = 1)	
**Rather yes**		2% (n = 1)	
**Undecided**		4% (n = 2)	
**Not really**		18% (n = 8)	
**Not at all**		73% (n = 33)	
**If discharged > 72 hours, preference for an earlier discharge?**		**Patients, % (n = 83)**	
**Yes**		14% (n = 12)	
**Rather yes**		17% (n = 14)	
**Undecided**		17% (n = 14)	
**Not really**		22% (n = 18)	
**Not at all**		30% (n = 25)	
**Satisfaction with medical instructions at 30 days**	**Early discharge**	**Standard discharge**	**p value**
**Very satisfied**	73% (n = 33)	54% (n = 45)	
**Satisfied**	18% (n = 8)	34% (n = 8)	
**Undecided**	2% (n = 1)	7% (n = 6)	p = 0.287
**Unsatisfied**	4% (n = 2)	2% (n = 2)	
**Very unsatisfied**	2% (n = 1)	2% (n = 2)	

## Discussion

This prospective cohort of ACS patients showed that early discharge was successfully achieved in one third of eligible patients (Zwolle index score ≤ 3 points) for whom there was no medical reason for prolonged hospitalization. Patients with a Zwolle index score > 3 points were older, suggesting that age and frailty are independent factors for determining the timing of hospital discharge in ACS patients. The most commonly reported clinical reason for prolonged hospitalization in patients with a Zwolle index score ≤ 3 points was a staged coronary procedure or need for prolonged cardiac monitoring in an intermediate care unit. Other issues were related to logistics, such as delay in complementary exams (e.g. echocardiography), titration of medication or admission/discharge during week-ends/nights. Patients in the eligible discharge group for early discharge had an excellent prognosis with a very low event rate at 30 days (minor bleeding) and were globally satisfied with the medical care provided and the LHS.

The Zwolle index score was developed and validated as a prognostic score to identify STEMI patients at low risk of complications who could be safely discharged within 72 hours.[3] ESC guidelines recommend the use of the Zwolle index score for the management of STEMI when selecting patients eligible for early discharge and for whom a schedule of systematic follow-up has been set up, for example in a cardiac rehabilitation setting.[2] The recent ESC guidelines for ACS management reported for the first time monitoring guidelines for an uncomplicated course of ACS; 24 hours of monitoring after PCI is recommended for an uncomplicated ACS, but can be prolonged beyond 24 hours in case of complications.[12]

We found that although two thirds of patients filled the criteria for early discharge according to the Zwolle index score, most of them had others medical reasons for prolonged hospital stay, such as revascularization in two steps during hospitalization.[13–15] Controversies persist regarding the application of revascularization for non culprit-lesions at the time of STEMI, with data suggesting of a potential benefit of complete revascularization during the index PCI procedure.[13,14,16] To our knowledge, no study has the assessed the cost-effectiveness of complete versus incomplete revascularization at the index PCI, nor of the strategies of complete revascularization during the initial hospital stay versus a staged procedure carried out at a later time. Given that the DRG reimbursement system is a strong incentive to shorten hospital stays, the use of complete revascularization during the index PCI might be an interesting strategy over a staged procedure. For the care-provider (hospital perspective), the staged procedure could be a financially valuable option in case of an hospitalization at distance from the index PCI, allowing for additional billing. Administrative data suggest that the LHS decreased in Switzerland with the introduction of DRG, without a decrease of the re-hospitalization rate at 90 days (+13.5%), although a causality cannot be proven.[6] Other leading documented reasons for prolonged hospitalization are the delay to perform recommended exams in the management of ACS patients, such as echocardiography. According to the recent ESC NSTE-ACS guidelines, echocardiography is recommended in all patients with ACS to assess cardiac function in perspective of risk stratification optimization of medical management as well as secondary prevention.

Previous studies have reported that few patients with ACS were discharged within 72 hours. In European countries, it is estimated that about 26–28% of potential eligible patients have an early discharge.[4,5] Compared to the United Stated, hospitalization for ACS is significantly longer in European countries, with the rate of early discharge being 60% in US compared to 15.9% in non-US countries. However, the readmission rate at 30 days is significantly higher in US than non-US countries, as is the presence of comorbidities (elderly, hypertension, diabetes, multiple vessels diseases and atrial fibrillation).[17] The Zwolle index score is a simple and useful method to select patients at low risk of complications early during hospitalization, according to age, PCI success and the presence of heart failure signs. The findings of our study suggest no increase in MACE, although results may limited by the relatively small sample size. Several previous studies suggested that early discharge was safe and feasible after uncomplicated PCI.[18–21] However, the design of a non-inferiority randomized controlled trial is a major challenge given the needed samples size and the possible « contamination effect » in health system research. The SAFE-depart trial performed a pilot randomized controlled study that was stopped after 50 patients due to slow recruitment (important exclusion criteria) and the length of stay was nearly similar in the early discharge versus standard discharge group.[4] The quality of care as defined by the use of recommended medication discharge was also similar across groups. Patient satisfaction also seemed high in the early discharge group, with a great majority of patients satisfied by the length of stay. It is well known that it is economically unattractive to manage low-risk patients for a prolonged hospital stay.[22]The following strategies have been reported to be successful in effective patient management: (1) identification of patients at low risk of complications (e.g. use of the Zwolle index score), (2) scheduling complementary exams as soon as possible (e.g. echocardiography), and (3) systematic cardiac rehabilitation follow-up after discharge.[2] An early appointment for cardiac rehabilitation has been associated with improved attendance and adherence to programs. The continuation and process of care after hospital discharge in a cardiac rehabilitation program is recommended to decrease the risk of cardiovascular mortality and morbidity.[23] In fact, in our study we systematically arranged for a follow-up appointment for our outpatient cardiac rehabilitation program, including free public transportation to encourage patients to come to the program within 10 days after hospital discharge.

Our study has some limitations. First, it describes a population of a single center in Switzerland, with a limited number of eligible patients (all benefited of coronary angiography), and might not be representative of other centers in Switzerland or in Europe. Interestingly, the proportion of patients who were discharged early was similar to that observed in other settings.[4,5] Secondary, we used the Zwolle index score for patients with ACS, and by default for STEMI patients as no score is recommended for ACS STEMI patients. Patients with NSTEMI (non ST elevation in ECG and positive troponin value) or unstable angina were included in the triage of patients using the Zwolle index score, as no score is recommended for ACS NSTEMI patients.[12] However, the recent ESC ACS guidelines have pointed out criteria, such as arrhythmia, unsuccessful PCI or heart failure, as high-risk for the monitoring and management of ACS patients. [12]

## Conclusion

Early discharge was successfully performed in one third of ACS patients at low risk of complications (Zwolle index score < 3), and appeared safe and highly appreciated by the patients. To reduce the LHS it’s important to organize quickly the cardiac rehabilitation and complementary exams. Further studies should describe the impact of shortening the length of hospitalization on clinical and patient reported outcomes, as well as the potential barriers for a widespread implementation of early discharge.

## Supporting Information

S1 Fig Distribution of the Zwolle index score among 370 patients with acute coronary syndromes confirmed by angiography. (TIF)

## References

[1] Spencer FA, Lessard D, Gore JM, Yarzebski J, Goldberg RJ (2004) Declining length of hospital stay for acute myocardial infarction and postdischarge outcomes: a community-wide perspective. Arch Intern Med 164: 733–740. 15078642 10.1001/archinte.164.7.733

[2] Steg PG, James SK, Atar D, Badano LP, Blomstrom-Lundqvist C, et al (2012) ESC Guidelines for the management of acute myocardial infarction in patients presenting with ST-segment elevation. Eur Heart J 33: 2569–2619. 10.1093/eurheartj/ehs215 22922416

[3] De Luca G, Suryapranata H, van 't Hof AW, de Boer MJ, Hoorntje JC, et al (2004) Prognostic assessment of patients with acute myocardial infarction treated with primary angioplasty: implications for early discharge. Circulation 109: 2737–2743. 15159293 10.1161/01.CIR.0000131765.73959.87

[4] Kotowycz MA, Cosman TL, Tartaglia C, Afzal R, Syal RP, et al (2010) Safety and feasibility of early hospital discharge in ST-segment elevation myocardial infarction—a prospective and randomized trial in low-risk primary percutaneous coronary intervention patients (the Safe-Depart Trial). Am Heart J 159: 117 e111–116. 20102876 10.1016/j.ahj.2009.10.024

[5] Barchielli A, Balzi D, Marchionni N, Carrabba N, Margheri M, et al (2007) Early discharge after acute myocardial infarction in the current clinical practice. Community data from the AMI-Florence Registry, Italy. Int J Cardiol 114: 57–63. 16712984 10.1016/j.ijcard.2006.01.006

[6] Busato A, von Below G (2010) The implementation of DRG-based hospital reimbursement in Switzerland: A population-based perspective. Health Res Policy Syst 8: 31 10.1186/1478-4505-8-31 20950481 PMC2973930

[7] Gencer Baris, G F, Sigaud Philippe, Meyer Philippe, Roffi Marco, Noble Stéphane and Mach François (2013) Emerging Concepts and Designing Effectiveness Research in the Process of Care of Patients Hospitalized with an Uncomplicated Acute Coronary Syndrome. Journal of Clinical Trials 3.

[8] Gencer B, Rodondi N, Auer R, Raber L, Klingenberg R, et al (2015) Reasons for discontinuation of recommended therapies according to the patients after acute coronary syndromes. Eur J Intern Med 26: 56–62. 10.1016/j.ejim.2014.12.014 25582072

[9] Gencer B, Auer R, Nanchen D, Raber L, Klingenberg R, et al (2015) Expected impact of applying new 2013 AHA/ACC cholesterol guidelines criteria on the recommended lipid target achievement after acute coronary syndromes. Atherosclerosis 239: 118–124. 10.1016/j.atherosclerosis.2014.12.049 25585031

[10] Auer R, Gencer B, Raber L, Klingenberg R, Carballo S, et al (2014) Quality of care after acute coronary syndromes in a prospective cohort with reasons for non-prescription of recommended medications. PLoS One 9: e93147 10.1371/journal.pone.0093147 24676282 PMC3968068

[11] Aujesky D, Roy PM, Verschuren F, Righini M, Osterwalder J, et al (2011) Outpatient versus inpatient treatment for patients with acute pulmonary embolism: an international, open-label, randomised, non-inferiority trial. Lancet 378: 41–48. 10.1016/S0140-6736(11)60824-6 21703676

[12] Roffi M, Patrono C, Collet JP, Mueller C, Valgimigli M, et al (2016) 2015 ESC Guidelines for the management of acute coronary syndromes in patients presenting without persistent ST-segment elevation: Task Force for the Management of Acute Coronary Syndromes in Patients Presenting without Persistent ST-Segment Elevation of the European Society of Cardiology (ESC). Eur Heart J 37: 267–315. 10.1093/eurheartj/ehv320 26320110

[13] Wald DS, Morris JK, Wald NJ, Chase AJ, Edwards RJ, et al (2013) Randomized trial of preventive angioplasty in myocardial infarction. N Engl J Med 369: 1115–1123. 10.1056/NEJMoa1305520 23991625

[14] Politi L, Sgura F, Rossi R, Monopoli D, Guerri E, et al (2010) A randomised trial of target-vessel versus multi-vessel revascularisation in ST-elevation myocardial infarction: major adverse cardiac events during long-term follow-up. Heart 96: 662–667. 10.1136/hrt.2009.177162 19778920

[15] Di Mario C, Mara S, Flavio A, Imad S, Antonio M, et al (2004) Single vs multivessel treatment during primary angioplasty: results of the multicentre randomised HEpacoat for cuLPrit or multivessel stenting for Acute Myocardial Infarction (HELP AMI) Study. Int J Cardiovasc Intervent 6: 128–133. 16146905 10.1080/14628840310030441

[16] Kelly DJ, McCann GP, Blackman D, Curzen NP, Dalby M, et al (2013) Complete Versus culprit-Lesion only PRimary PCI Trial (CVLPRIT): a multicentre trial testing management strategies when multivessel disease is detected at the time of primary PCI: rationale and design. EuroIntervention 8: 1190–1198. 10.4244/EIJV8I10A183 23425543

[17] Kociol RD, Lopes RD, Clare R, Thomas L, Mehta RH, et al (2012) International variation in and factors associated with hospital readmission after myocardial infarction. JAMA 307: 66–74. 10.1001/jama.2011.1926 22215167

[18] Melberg T, Jorgensen M, Orn S, Solli T, Edland U, et al (2015) Safety and health status following early discharge in patients with acute myocardial infarction treated with primary PCI: a randomized trial. Eur J Prev Cardiol 22: 1427–1434. 10.1177/2047487314559276 25398704

[19] Jones DA, Rathod KS, Howard JP, Gallagher S, Antoniou S, et al (2012) Safety and feasibility of hospital discharge 2 days following primary percutaneous intervention for ST-segment elevation myocardial infarction. Heart 98: 1722–1727. 10.1136/heartjnl-2012-302414 23053711

[20] Jirmar R, Widimsky P, Capek J, Hlinomaz O, Groch L (2008) Next day discharge after successful primary angioplasty for acute ST elevation myocardial infarction. An open randomized study "Prague-5". Int Heart J 49: 653–659. 19075481 10.1536/ihj.49.653

[21] Azzalini L, Sole E, Sans J, Vila M, Duran A, et al (2015) Feasibility and safety of an early discharge strategy after low-risk acute myocardial infarction treated with primary percutaneous coronary intervention: the EDAMI pilot trial. Cardiology 130: 120–129. 10.1159/000368890 25612789

[22] Laarman GJ, Dirksen MT (2010) Early discharge after primary percutaneous coronary intervention. Heart 96: 584–587. 10.1136/hrt.2009.171363 19778921

[23] Smith SC Jr., Benjamin EJ, Bonow RO, Braun LT, Creager MA, et al (2011) AHA/ACCF Secondary Prevention and Risk Reduction Therapy for Patients with Coronary and other Atherosclerotic Vascular Disease: 2011 update: a guideline from the American Heart Association and American College of Cardiology Foundation. Circulation 124: 2458–2473. 10.1161/CIR.0b013e318235eb4d 22052934

