# Supplementary figures and images for: Early Discharge in Low-Risk Patients Hospitalized for Acute Coronary Syndromes: Feasibility, Safety and Reasons for Prolonged Length of Stay
Source: PLoS One. 2016 Aug 23;11(8):e0161493. doi: 10.1371/journal.pone.0161493 (PMC4994963; doi:10.1371/journal.pone.0161493)

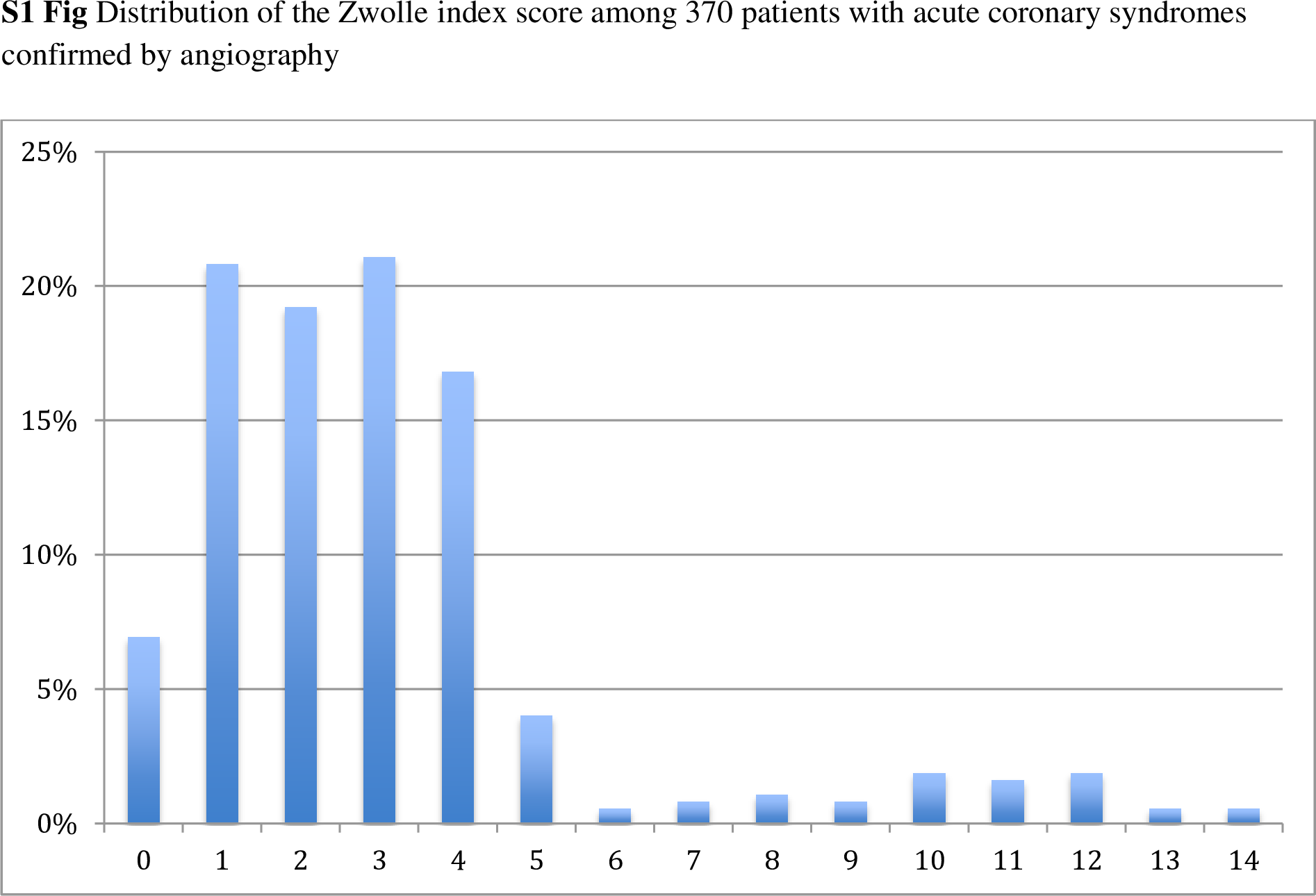

Supplement: S1 Fig — (TIF) [file pone.0161493.s001.tif]
